# Hydrogen Peroxide-induced Cell Death in Mammalian Cells

**DOI:** 10.33696/signaling.2.052

**Published:** 2021

**Authors:** Tamutenda Chidawanyika, Surachai Supattapone

**Affiliations:** 1 Department of Biochemistry, Geisel School of Medicine at Dartmouth, Hanover, NH 03755, USA; 2 Department Medicine, Geisel School of Medicine at Dartmouth, Hanover, NH 03755, USA

**Keywords:** Hydrogen peroxide, Riboflavin, Cell death, Aquaporin, Leukocyte

## Abstract

Hydrogen peroxide (H_2_O_2_) is an important intra- and extra-cellular signaling molecule that can determine cell fate. At low concentrations, H_2_O_2_ plays roles in proliferation, immunity, and metabolism. Cellular exposure to higher non-physiologic concentrations of H_2_O_2_ can result in oxidative stress. If the stress is not alleviated, cell death can ensue. In the past, few studies were done to study the key mediators of H_2_O_2_-induced cell death. The advancement of genetic screening technology with CRISPR/Cas9 tools has allowed for in depth genome-wide studies to identify key mediators in different cell types. Here, we briefly explore the role of H_2_O_2_ in the cell and the essential mediators of H_2_O_2_-induced cell death with a focus on riboflavin, an unexpected essential mediator of H_2_O_2_-induced cell death.

## H_2_O_2_ Exposure as a Form of Oxidative Stress

Sies first described oxidative stress in 1985 as the intracellular process that occurs when the balance between reactive oxygen species (ROS) and the cellular antioxidant defense system is perturbed, and the levels of the ROS exceed cellular homeostatic concentrations [1–3]. ROS such as hydrogen peroxide (H_2_O_2_), superoxide radical $\left({{\text{O}}_{\text{2}}}^{•-}\right)$, and hydroxyl radical $\left({\text{OH}}^{•}\right)$ are chemically reactive with various molecules in cells such as DNA, proteins, lipids, and sugars [4,5]. Among the known ROS, H_2_O_2_ is thought to be one of the less reactive and more stable species, and this quality allows H_2_O_2_ to behave as an important intra- and inter-cellular signaling molecule, and as a cell fate determinant [6]. At physiologic concentrations, H_2_O_2_ influences cellular processes including differentiation, proliferation, metabolism, and immunity [6]. However, when H_2_O_2_ levels increase beyond homeostatic concentrations, oxidative stress occurs and if it is not alleviated through antioxidant responses, cell death can occur [6].

## Antioxidant Responses to H_2_O_2_-induced Oxidative Stress

To alleviate oxidative stress, cells launch a wide range of antioxidant responses that are designed to scavenge ROS. Here, we discuss two antioxidant response systems that are involved in the alleviation of H_2_O_2_-induced oxidative stress, namely, the nuclear factor erythroid 2-related factor 2 (Nrf2) axis, and riboflavin, also known as vitamin B2.

Antioxidant responses are composed of complex signaling pathways that can be transcriptionally regulated by redox-sensitive transcription factors such as Nrf2, a master regulator of antioxidant response in cells [7]. Under normal conditions, Nrf2 is bound to kelch-like ECH associated protein 1 (KEAP1) in the cytoplasm [8]. KEAP1 is an adaptor of Cul3-based E3 ubiquitin ligase and therefore, it targets Nrf2 for proteasomal degradation [9]. During oxidative stress, the cysteine residues on KEAP1 are oxidized and Nrf2 is released from its association with KEAP1 [8,9]. Nrf2 then translocates to the nucleus, where it binds to the antioxidant response element (ARE) to regulate a plethora of genes (almost 200 genes) that are involved in cellular defense against oxidative stress [9]. Some of the activated proteins that are involved in reducing H_2_O_2_ to H2O and O2 include catalase, peroxiredoxins, and glutathione peroxidases (GPXs) [9].

Antioxidants can also exist in the form of vitamins such as vitamin C, vitamin E, carotenoids, and riboflavin [10]. In the case of riboflavin, this vitamin has been shown to have a protective effect against lipid peroxidation and tissue reperfusion injury, both of which are due to oxidative stress [10]. The mechanism of action of this protection has been shown to be through riboflavin’s role in the glutathione redox cycle, and through the conversion of riboflavin from its reduced form to its oxidized form during oxidative stress [10]. If a cell can increase its antioxidant capacity, homeostasis will be restored and the cell will survive. However, if the antioxidant defense systems fail, the cell will die.

## Mechanisms and Essential Mediators of H_2_O_2_-induced Cell Death

H_2_O_2_ has been found to cause cell death through apoptosis, autophagy, necrosis, and ferroptosis [11–15]. Cell type and the severity of the oxidative stress to which a cell is exposed influences the cell death modality employed [11–13]. Hence, the concentration or duration of exposure of a cell type to H_2_O_2_ can also affect the mechanism of cell death. Caspase inactivation due to oxidative stress has been implicated as the reason why H_2_O_2_-induced cell death switches from apoptosis to other cell death modalities [16].

The use of CRISPR/Cas9-based genetic screens has advanced our biological knowledge and understanding of how exposure to extracellular H_2_O_2_ causes death in different cell types. It is important to note that screening parameters, including severity of exposure to H_2_O_2_ and cell type, vary for different screens. This factor not only affects the cell death modality employed by the cells, but it also affects the nature of the identified mediators of H_2_O_2_-induced cell death. Interestingly, the mediators have included genes involved in redox biology, riboflavin metabolism, iron homeostasis, vesicle trafficking, and the peroxisomal import pathway [17,18]. Here, we briefly discuss some of the mediators that are involved in redox biology, namely cytochrome P450 reductase (POR), retinol saturase (RETSAT), and KEAP1 [17,18]. The mediators involved in riboflavin metabolism, namely riboflavin kinase (RFK), flavin adenine dinucleotide synthetase 1 (FAD synthase), and SLC52A2 which is a riboflavin transporter with the highest affinity for riboflavin, are discussed in more depth [17,18]. The mechanism of cell death employed by POR, RETSAT, KEAP1 and SLC52A2 in HAP1 cells is iron-dependent, suggesting that these proteins mediate H_2_O_2_-induced cell death through ferroptosis in HAP1 cells [18]. It is important to note that these same proteins may mediate H_2_O_2_-induced cell death in other cell types through other mechanisms of cell death such as apoptosis.

POR, RETSAT, and KEAP1 are involved in redox biology, and they have been identified and validated as essential mediators of cell death in multiple independent screens against H_2_O_2_. POR was first identified for its role in oxidative stress-induced cell death in a paraquat CRISPR/Cas9 screen in Jurkat cells [19]. It’s role in sensitization of cells to ROS-induced cell death, specifically H_2_O_2_, was later confirmed in CRISPR/Cas9 screens in K562 cells, HeLa cells, and in HAP1 cells [17]. In NIH/3T3 cells, a mouse embryonic fibroblast cell line, RETSAT was initially identified as a mediator of H_2_O_2_-induced cell death using an RNAi-based genetic screen [20,21]. CRISPR/Cas9 genome wide screens in human cell lines, K562 cells, HeLa cells, and HAP1 cells, confirmed the importance of RETSAT as an essential mediator of H_2_O_2_-induced cell death in human cell lines [17]. As an inhibitor of Nrf2 antioxidant activity, it is not surprising that KEAP1 was identified in CRISPR/Cas9 screens in K562, HeLa, and HAP1 cells [17,18].

## Riboflavin-mediated H_2_O_2_-induced Cell Death

The identification of mediators involved in riboflavin metabolism as being involved in H_2_O_2_-induced cell death is of particular interest since historically, riboflavin has been regarded as an antioxidant, rather than an agent that promotes oxidative stress [10]. The screens in K652 and HeLa cells identified RFK and FAD synthase, while the screen in HAP1 cells identified SLC52A2 [17,18]. The identification of distinct mediators that are involved in riboflavin metabolism in the three screens is likely due to the use of different screening conditions with varying H_2_O_2_ concentrations, as well as different cell types. In the screens against K652 cells and HeLa cells, H_2_O_2_ concentrations were based on the concentration that resulted in survival of 50% of the cells [17]. However, in the screen against HAP1 cells, H_2_O_2_ concentrations were based on the concentration that resulted in survival of less than 1% of the cells [18]. Of note, the fact that riboflavin mediators were identified in three distinct screens with different conditions, shows the importance of riboflavin in H_2_O_2_-induced cell death. This importance was also highlighted when *SLC52A2* knockout cells and HAP1 cells that were grown in riboflavin-depleted media showed resistance to H_2_O_2_-induced cell death, while *SLC52A2* competent cells and cells grown in riboflavin-containing media did not survive H_2_O_2_ treatment [18].

**Figure 1:**
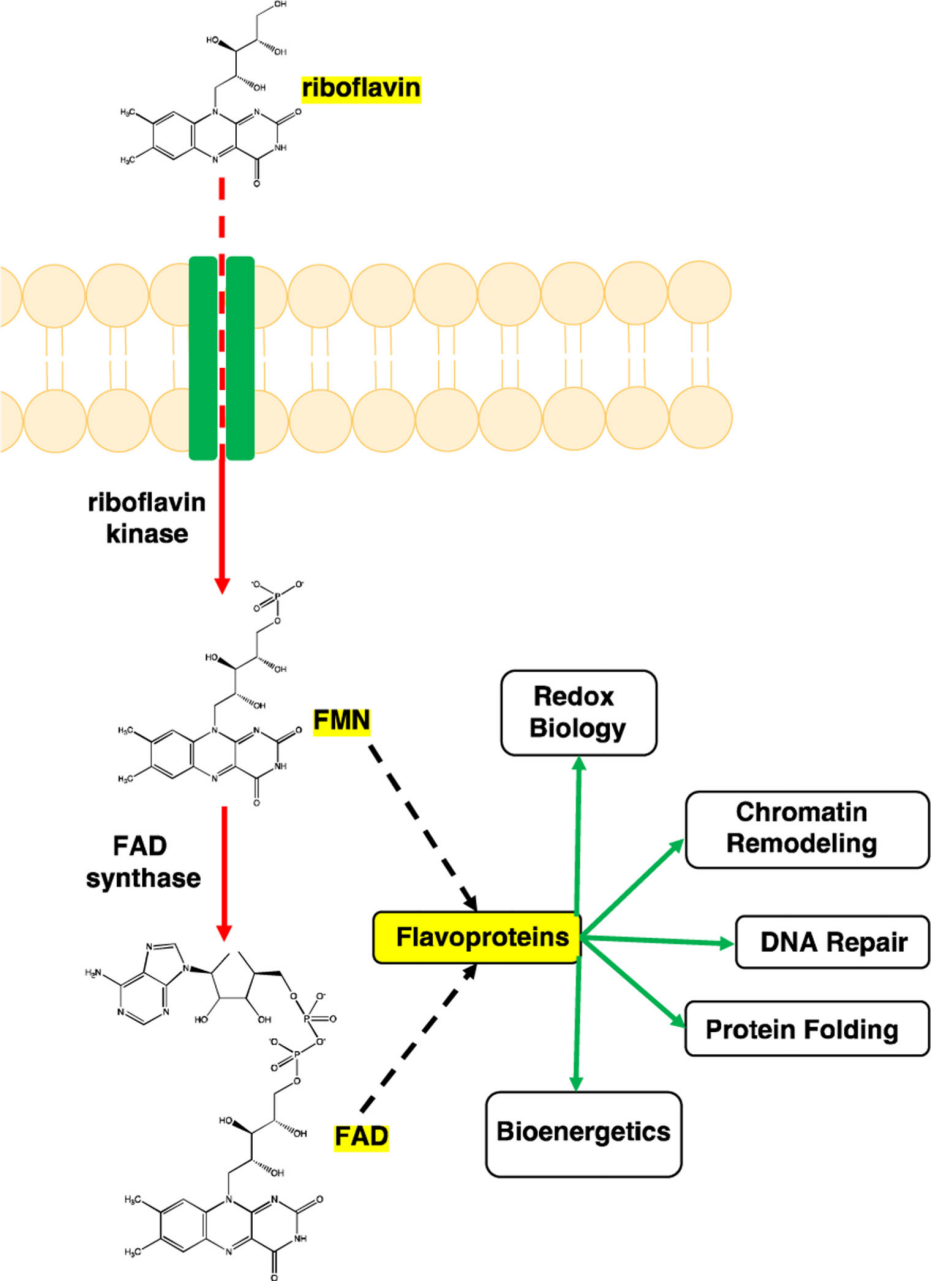
Riboflavin is converted to its biologically active metabolites, flavin mononucleotide (FMN) and flavin adenine dinucleotide (FAD). Riboflavin enters the cell through riboflavin transporters (SLC52A1, SLC52A2, or SLC52A3) and it is converted to FMN by riboflavin kinase. FAD synthase converts FMN to FAD. FMD and FAD are important cofactors for flavoproteins that are involved in multiple cellular processes. Image generated using ChemDraw (PerkinElmer).

Riboflavin (vitamin B2) is important for normal cellular function. Riboflavin enters cells through riboflavin transporters (SLC52A1, SLC52A2, or SLC52A3) and once in the cell, riboflavin kinase (RFK) converts riboflavin to flavin mononucleotide (FMN), which is then converted to flavin adenine dinucleotide (FAD) by FAD synthase [22–24]. FMN and FAD are the metabolically active forms of riboflavin, and they are important cofactors for many flavoproteins such as POR and RETSAT [25,26]. These flavoproteins are involved in many cellular processes such as energy metabolism, redox biology, DNA repair, and chromatin remodeling (Figure 1) [27]. Riboflavin’s known role as an antioxidant has favored its use as a protective agent against oxidative stress, particularly since FAD is an important cofactor for glutathione reductase which is a key cellular antioxidant [28]. Recent work from CRISPR/Cas9-based screens in K562, HeLa, and HAP1 cells, however, has shown that riboflavin has a pro-oxidant and cytotoxic role in oxidative stress induced by H_2_O_2_ [17].

A potential explanation for this switch in riboflavin’s role in redox metabolism is that H_2_O_2_ concentrations influence riboflavin’s role in redox biology. In this scenario, at lower H_2_O_2_ concentrations, riboflavin serves as an antioxidant to maintain cellular homeostasis. Conversely, at higher H_2_O_2_ concentrations, riboflavin serves as a pro-oxidant to mediate cell death. A simple explanation for riboflavin’s role in cell death at higher concentrations of H_2_O_2_ could be that the higher H_2_O_2_ concentrations oxidize riboflavin to form a toxic species that can cause cell death. However, when HAP1 cells were treated with riboflavin that had been pre-treated with H_2_O_2_ to generate an oxidized form of riboflavin, the oxidized riboflavin was not cytotoxic to HAP1 cells, making this explanation unlikely [18]. Further experimentation will be necessary to explore riboflavin’s dual role in redox biology.

## Mechanisms of Riboflavin-mediated H_2_O_2_-induced Cell Death

Through fluorescence-based experiments in HAP1 cells, riboflavin was shown to mediate H_2_O_2_ entry into HAP1 cells [18]. This role in regulation of H_2_O_2_ cell entry is a potential mechanism of action for riboflavin in H_2_O_2_-induced cell death. In riboflavin-depleted media, H_2_O_2_ cell entry into HAP1 cells was significantly less than in cells grown in riboflavin-competent media [18]. Without riboflavin, H_2_O_2_ would not be able to have intracellular access in HAP1 cells [18]. H_2_O_2_ cell entry is known to be mediated by a group of integral membrane proteins called aquaporins [29,30]. Aquaporins also mediate water, glycerol, and urea transport across cell membranes [31]. Aquaporin-based transport can therefore be evaluated by monitoring changes in cellular volume as a result of osmotic stress [31]. Osmotic stress experiments showed that volume regulation during osmotic stress depends on riboflavin in HAP1 cells, suggesting that riboflavin’s regulation of H_2_O_2_ cell entry is likely through interactions with an aquaporin [18]. This hypothesis is supported by the fact that AQP3 was identified as a mediator of H_2_O_2_-induced cell death in the screens in K562 and HeLa cells [17]. Further experimentation would be necessary to explore and confirm the proposed relationship between riboflavin, AQP3, and H_2_O_2_.

Interestingly, fluorescence-based experiments in retinal pigment epithelium and HEK293 cells did not show riboflavin-dependent H_2_O_2_ cell entry [18], suggesting that riboflavin’s function in H_2_O_2_ cell entry is cell type specific. HAP1 cells are leukocytes that were derived from KBM-7 cells, which were isolated from a patient with chronic myelogenous leukemia (CML) in blast crisis [32,33]. When leukocytes are in riboflavin-deficient environments, their phagocytic activity decreases [34]. After leukocytes phagocytose microbes, the respiratory burst which can use H_2_O_2_, kills the microbes [35–37]. One potential reason why H_2_O_2_ cell entry is riboflavin-dependent in HAP1 cells is that these leukocytes use the riboflavin status of their environment as a measure of their own energy levels, and therefore of their ability to participate in the respiratory burst. If riboflavin levels are adequate, microbe phagocytosis can occur, H_2_O_2_ is allowed into the cell, and the respiratory burst can proceed. Riboflavin-based experimentation in other cells lines will be necessary to explore this hypothesis further.

Another potential mechanism of action for riboflavin in H_2_O_2_-induced cell death is through flavoproteins such as POR and RETSAT. Given that POR and RETSAT have FAD and FMN binding sites that permit participation of these proteins in redox biology, it is entirely possible that riboflavin’s role in H_2_O_2_-induced cell death is based on the interactions of its metabolites with POR and RETSAT [25,26]. Further experimentation would be needed to explore this hypothesis.

## Conclusion

**Figure 2:**
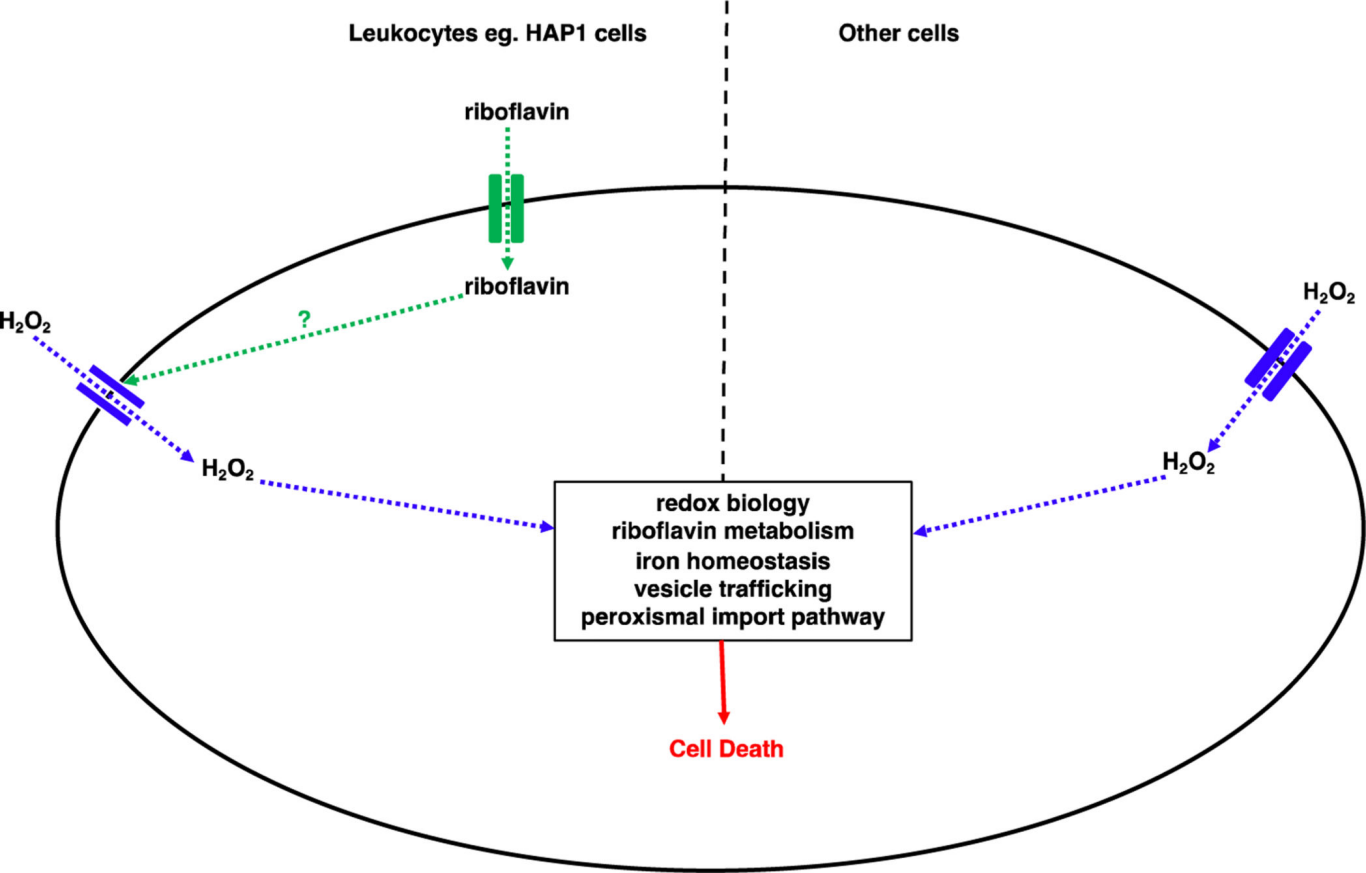
Models for H_2_O_2_-induced cell death in cells of leukocytic lineage and in other cells. In cells of leukocytic lineage such as HAP1 cells, H_2_O_2_ cell entry is riboflavin-mediated. H_2_O_2_ enters the cell through an aquaporin and through mediators of redox biology, riboflavin metabolism, iron homeostasis, vesicle trafficking, and the peroxisomal import pathway, cell death occurs. In cells that are not of leukocytic lineage, riboflavin does not mediate H_2_O_2_ cell entry.

The mechanism of cell death and the mediators employed in H_2_O_2_-induced cell death depend on the severity and degree of exposure to H_2_O_2_, as well as the cell type. Based on the genetic screens against H_2_O_2_ so far, hypothetical models of how H_2_O_2_ induces cell death in leukocytes and other cell types are shown (Figure 2). Of particular interest and in need of further exploration, is riboflavin’s mediation of H_2_O_2_-induced cell death through regulation of H_2_O_2_ cell entry in leukocytes.
